# Comparative study on antibacterial characteristics of the multiple liver expressed antimicrobial peptides (LEAPs) in teleost fish

**DOI:** 10.3389/fimmu.2023.1128138

**Published:** 2023-02-20

**Authors:** Xun Liu, Ya-Zhen Hu, Yi-Ru Pan, Jia Liu, You-Bo Jiang, Yong-An Zhang, Xu-Jie Zhang

**Affiliations:** ^1^ State Key Laboratory of Agricultural Microbiology, Hubei Hongshan Laboratory, Engineering Research Center of Green Development for Conventional Aquatic Biological Industry in the Yangtze River Economic Belt, Ministry of Education, College of Fisheries, Huazhong Agricultural University, Wuhan, China; ^2^ Guangdong Laboratory for Lingnan Modern Agriculture, Guangzhou, China

**Keywords:** rainbow trout, grass carp, LEAP-1, LEAP-2, expression pattern, antibacterial activity

## Abstract

Antimicrobial peptides are important components of the host innate immune system, forming the first line of defense against infectious microorganisms. Among them, liver-expressed antimicrobial peptides (LEAPs) are a family of antimicrobial peptides that widely exist in vertebrates. LEAPs include two types, named LEAP-1 and LEAP-2, and many teleost fish have two or more *LEAP-2s*. In this study, *LEAP-2C* from rainbow trout and grass carp were discovered, both of which are composed of 3 exons and 2 introns. The antibacterial functions of the multiple LEAPs were systematically compared in rainbow trout and grass carp. The gene expression pattern revealed that rainbow trout and grass carp *LEAP-1*, *LEAP-2A*, *LEAP-2B* and/or *LEAP-2C* were differentially expressed in various tissues/organs, mainly in liver. After bacterial infection, the expression levels of *LEAP-1*, *LEAP-2A*, *LEAP-2B* and/or *LEAP-2C* in the liver and gut of rainbow trout and grass carp increased to varying degrees. Moreover, the antibacterial assay and bacterial membrane permeability assay showed that rainbow trout and grass carp LEAP-1, LEAP-2A, LEAP-2B and LEAP-2C all have antibacterial activities against a variety of Gram-positive and Gram-negative bacteria with varying levels through membrane rupture. Furthermore, cell transfection assay showed that only rainbow trout LEAP-1, but not LEAP-2, can lead to the internalization of ferroportin, the only iron exporter on cell surface, indicating that only LEAP-1 possess iron metabolism regulation activity in teleost fish. Taken together, this study systematically compared the antibacterial function of LEAPs in teleost fish and the results suggest that multiple LEAPs can enhance the immunity of teleost fish through different expression patterns and different antibacterial activities to various bacteria.

## Introduction

Antimicrobial peptides (AMPs) are widely found in organisms and are considered to be the first line of host defense against pathogens (1, 2). With the increasing resistance of bacteria to traditional antibiotics, AMPs have gradually become one of the important alternatives to antibiotics (3, 4). There are two main antibacterial mechanisms of AMPs: direct sterilization and immune regulation, and the sterilization mechanism can be divided into membrane targeting and non-membrane targeting (5). In addition to their broad-spectrum antibacterial activities, AMPs can also inhibit fungi, viruses and parasites (6). Compared with other species, some AMPs derived from fish, shrimp, crabs and other aquatic animals have unique structures and functions (7, 8).

Liver-expressed antimicrobial peptide (LEAP) is a class of antibacterial peptides expressed and secreted by liver, including LEAP-1 and LEAP-2. Both LEAP-1 and LEAP-2 can be separated and purified from human plasma (9, 10). *LEAP-1* exists as a single gene in the vast majority of mammals, which is highly expressed in the liver, followed by heart and brain (9). Because of its dual functions of antibacterial and iron regulation, LEAP-1 is also known as hepcidin. LEAP-1 mainly binds to ferroportin (Fpn), the only known iron exporter on the surface of cells (11), to facilitate its internalization, thus affecting the absorption and release of iron ion in iron storage cells (12–14). Fish have two hepcidin isoforms, hamp1 and hamp2; however, hamp1 is found to exist in both ray-finned and lobe-finned fish, while hamp2 is only found to exist in ray-finned fish, and the tissue distribution of hepcidin in most teleost fish is polymorphic (15–17).

Mammalian *LEAP-2* is a single gene, which is mainly expressed by the liver, and to a certain extent by other tissues (18). For example, mouse *LEAP-2* is mainly expressed in the liver and small intestine (18). Studies have shown that the mature peptide 38-77 of human LEAP-2 has different killing effects on both Gram-positive and Gram-negative bacteria, while its cleaved form 44-77 loses antibacterial activity but participates in the blood circulation of the body (19). Therefore, LEAP-2 may have other physiological functions besides sterilization. However, unlike mammals with a single *LEAP-2*, three *LEAP-2s* have been discovered in fish species, including *LEAP-2A*, *LEAP-2B* and *LEAP-2C* (20–23), and the tissue expression patterns in different fish species are also different (22, 24). Teleost LEAP-2 can enhance the bacterial killing efficiency of monocytes/macrophages (25, 26) and has a synergistic effect with antibiotics on the killing of drug-resistant bacteria (27).

So far, systematic comparative studies on the antibacterial functions of LEAPs are still blank. Therefore, this study was devoted to analyzing the antibacterial functions of LEAPs and conducting a systematic comparative study to lay a theoretical foundation for their use as feed additives to prevent bacterial diseases in fish.

## Materials and methods

### Experimental fish and cells

Rainbow trout (*Oncorhynchus mykiss*) (30 ± 5 g) and grass carp (*Ctenopharyngodon idella*) (200 ± 20 g) were purchased from Dujiang Dam Rainbow Trout Farm (Chengdu, China) and Xiantao Hatchery (Xiantao, China) respectively. They were maintained and acclimated to the laboratory conditions for at least two weeks before experiments. All animal experiments in this study were approved by the Committee on the Ethics of Animal Experiments at Huazhong Agricultural University.

Human embryonic kidney 293T (HEK293T) cells were cultured in 5% CO_2_ at 37°C. Dulbecco’s modified Eagle’s medium (DMEM) (HyClone) was supplemented with 10% fetal bovine serum (FBS) (Gibco), 100 g/mL penicillin (Sigma-Aldrich), and 100 g/mL streptomycin (Sigma-Aldrich).

### Searching, identification, and localization of *LEAPs* in rainbow trout and grass carp genome

The chromosome-level genome of grass carp was assembled by our laboratory and deposited in the NCBI BioProjects with the accession number PRJNA745929 (28). The published fish *LEAPs*, especially zebrafish *LEAPs*, were used to search against the grass carp genome using the Basic Local Alignment Search Tool (BLAST). Interestingly, in addition to *LEAP-2A* and *LEAP-2B*, *LEAP-2C* was found in grass carp. The grass carp *LEAP-2C* was cloned and submitted to the GenBank database (https://www.ncbi.nlm.nih.gov/genbank/) under the accession number OQ026323. Similarly, the rainbow trout *LEAP-2C* was cloned and submitted to the GenBank database under the accession number GQ870279.1.

### Sequence alignment and phylogenetic analysis

The signal peptide of the deduced amino acid sequences was predicted using the SignalP 5.0 Server (https://services.healthtech.dtu.dk/service.php?SignalP-5.0). The *LEAPs* gene organizations, including exon, intron and UTR were determined by aligning the cDNA sequences with the gene sequences. The protein sequence identity was calculated using the BioEdit software (version 7.0.9). Multiple sequence alignment was conducted with the ClustalX program (version 3.0), and phylogenetic tree was constructed based on the alignments using the neighbour-joining method with 1000 bootstrap times using the MEGA program (version 4.1). All the sequences used for multiple sequence alignment and phylogenetic analysis were listed in **Table 1**.

**Table 1:** LEAP sequences used in this study.

Species	Protein	GenBank accession no.
*Homo sapiens*	LEAP-1	NP_066998.1
*Oryctolagus cuniculus*	LEAP-1	XP_008247717.1
*Mus musculus*	LEAP-1	NP_115930.1
*Strigops habroptila*	LEAP-1	XP_030330703.1
*Xenopus laevis*	LEAP-1	XP_018097982.2
*Zootoca vivipara*	LEAP-1	XP_034976619.1
*Oncorhynchus mykiss*	LEAP-1	ADU85830.1
*Ctenopharyngodon idella*	LEAP-1	AEZ51835.1
*Danio rerio*	LEAP-1	NP_991146.1
*Salmo salar*	LEAP-1	NP001134321.1
*Scophthalmus maximus*	LEAP-1	AAX92670.1
*Dicentrarchus labrax*	LEAP-1	KJ890391.1
*Homo sapiens*	LEAP-2	AJ306405.1
*Oryctolagus cuniculus*	LEAP-2	NP_001164729.1
*Mus musculus*	LEAP-2	AJ409055.1
*Gallus gallus*	LEAP-2	AAS99322.1
*Strigops habroptila*	LEAP-2	XP_030360362.1
*Xenopus laevis*	LEAP-2	NC_054375.1
*Zootoca vivipara*	LEAP-2	XP_034961285.1
*Oncorhynchus mykiss*	LEAP-2A	AAR11766.1
*Oncorhynchus mykiss*	LEAP-2B	AAR11767.1
*Oncorhynchus mykiss*	LEAP-2C	GQ870279.1
*Ctenopharyngodon idella*	LEAP-2A	ACR54299.1
*Ctenopharyngodon idella*	LEAP-2B	AOG20830.1
*Ctenopharyngodon idella*	LEAP-2C	OQ026323
*Danio rerio*	LEAP-2A	BC162807.1
*Danio rerio*	LEAP-2B	AL918619.1
*Danio rerio*	LEAP-2C	NP_001373333.1

### The mRNA expression of *LEAPs* in rainbow trout and grass carp tissues

Four healthy rainbow trout and grass carp were anesthetized with MS222 (1:10000), then the blood was removed from the body by cardiac perfusion using phosphate-buffered saline (PBS; pH 7.4; Gibco). The head kidney, spleen, gut, gill, skin, liver, heart, and muscle were collected, and the total RNA was extracted using the TRIzol Reagent (Takara). The cDNA was synthesized using the PrimeScript™ RT Reagent Kit contains gDNA Eraser (Takara). The mRNA expression levels of *LEAPs* were detected by quantitative real-time PCR (qPCR) using the CFX Connect™ Real-Time System (Bio-Rad). The primers used are listed in **Table 2**. The reaction mixture (20 μl) contained 1 μl cDNA, 10 μl SsoAdvanced™ SYBR Green Supermix (Bio-Rad), 1 μl forward primer (10 μM each) and 1 μl reverse primer (10 μM each). The amplification program was as follows: 95°C for 5 min, 45 cycles of amplification (95°C for 5 s and 60°C for 30 s), and then 65°C for 5 s. The tissue expression levels of *LEAPs* were determined using 2^−ΔCt^ method with *β-actin* as the internal reference.

**Table 2:** Primers used in this study.

Name	Sequence (5’→3’)	Application
rtLEAP-1-QF	CATTTCAGGTTCAAGCGTCAGA	Real-time PCR
rtLEAP-1-QR	ATTTGCAGCAGAAGCCACAGC	Real-time PCR
rtLEAP-2A-QF	CTGCCAGCCCTGTTCCATCT	Real-time PCR
rtLEAP-2A-QR	CATCCGCTTCAGTGCCCTCT	Real-time PCR
rtLEAP-2B-QF	TGGTGGCTCTGATTCTTATGCA	Real-time PCR
rtLEAP-2B-QR	TCATGCGGGTTCTCCGTTCC	Real-time PCR
rtLEAP-2C-QF	GAATACTCTGAAGCCCGTTGG	Real-time PCR
rtLEAP-2C-QR	ATTTGGTCCCGCACTCGTCG	Real-time PCR
gcLEAP-1-QF	ACAGCAGGAGCAGGATGAGC	Real-time PCR
gcLEAP-1-QR	TATCCACAGCCTTTGTTACGAC	Real-time PCR
gcLEAP-2A-QF	TGGTGATTGTCCAGCAGGTGA	Real-time PCR
gcLEAP-2A-QR	GTAATGGTTCTGGCAGTAGGC	Real-time PCR
gcLEAP-2B-QF	GTATCTACTGTGCCATTAGCGA	Real-time PCR
gcLEAP-2B-QR	GACATTCGTATCTTGCGGTGC	Real-time PCR
gcLEAP-2C-QF	CGGCACCCGTAGATACTGAC	Real-time PCR
gcLEAP-2C-QR	GTGTTCCATCGCCATAGTAAAG	Real-time PCR
rtβ-actin-QF	ACAGGTCATCACCATCGGCA	Real-time PCR
rtβ-actin-QR	GGTCTCGTGGATACCGCAAG	Real-time PCR
gcβ-actin-QF	AGCCATCCTTCTTGGGTATG	Real-time PCR
gcβ-actin-QR	GGTGGGGCGATGATCTTGAT	Real-time PCR
pEGFP-Fpn-F	CGGGGTACCGATGGATAACGCGGGACCTAAG	Expression
pEGFP-Fpn-R	TCCCCCCGGGACACCGTGGTGGGAAGCAA	Expression

The restriction enzyme sites are underlined. F, forward; R, reverse.

### Bacterial infection

To detect the immune responses of LEAPs during infection, rainbow trout and grass carp were intraperitoneally injected with 100 µL *Aeromonas salmonicida* BG1 (29) suspension culture (1×10^7^ CFU/mL) and 200 µL *Aeromonas hydrophila* XS91-4-1 (30) suspension culture (8×10^6^ CFU/mL) respectively, while the control fish were intraperitoneally injected with PBS instead after anesthetized with MS222 (1:10000). At 12 h, 1 d, 3 d, 5 d, and 7 d post-injection, the liver and gut of four individuals were sampled from each group. After the RNA extraction and cDNA synthesis, the expression levels of *LEAPs* in infected and control fish were determined by qPCR. The expression changes of *LEAPs* after infection were calculated using the 2^−ΔΔCt^ method, with *β-actin* as the internal reference.

### Peptide synthesis

The mature peptides of rainbow trout and grass carp LEAPs were synthesized by GL Biochem Ltd. The purity was confirmed to be higher than 95% by HPLC and MALDI-TOF mass spectroscopy. All peptides were stored at -80°C for later use.

### Antibacterial activity assay

Six Gram-negative bacterial strains (*Escherichia coli* ATCC25922, *A. hydrophila* XS91-4-1, *A. salmonicida* BG1, *A. sobria* CR79-1-1, *Edwardsiella ictaluri* HSN-1, and *Vibrio fluvialis* WY91-24-3) and two Gram-positive bacterial strains (*Micrococcus luteus* ATCC10240 and *Streptococcus agalactiae* ATCC13813) were used in the antibacterial activity assay. Radial-diffusion assay (RDA) was conducted as previously described (31, 32). Briefly, 5 mL underlay agarose gel containing 0.03% (wt/vol) TSB, 1% (wt/vol) low electroendosmosis (EEO) agarose (Aladdin), 0.02% (vol/vol) Tween 20 (Amresco), and 4×10^6^ CFU bacteria was added into each sterile petri dish (90 mm diameter). After the solidification of the agarose, 4 mm diameter wells were punched in the agar using a sterile steel borer. Then 6 μL peptide (125 μM) was added to each well, and sterile water was added as control. Agar plates were incubated for 3 h at the optimum bacterial growth temperature (28°C for *A. hydrophila*, *A. salmonicida*, *A. sobria* and *E. ictaluri* or 37°C for *E. coli, V. fluvialis, M. luteus* and *S. agalactiae*) to diffuse the peptides. Each plate was added 5 mL sterilized overlay agarose gel (containing 6% TSB and 1% low EEO agarose) and incubated for 24 h at the optimum bacterial growth temperature. The diameter of the clearance zone around each well was measured.

### Bacterial membrane permeability assay

The membrane permeability of bacteria after the treatment of peptides was assessed by flow cytometry as previously described (33) with minor modifications. In brief, *V. fluvialis* WY91-24-3 were cultured to mid-logarithmic phase, then washed twice and resuspended to 10^6^ CFU/ml with 10 mM NaPB (pH 7.4). Thereafter, 40 μl of peptides diluted in NaPB was added to 160 μl of *V. fluvialis* to give a final concentration of 8 μM. LL37 and NaPB was used as the positive and negative control, respectively. After being incubated at 37°C for 1 h, propidium iodide (PI; Sigma-Aldrich) was added to the bacterial suspension at a final concentration of 9 μM. The PI positive bacterial cells were detected using the flow cytometer FACSVerse (BD Biosciences) at 3,000 events and the data were analyzed using the FlowJo software v10 (Tree Star).

### Plasmid construction and transfection

The *Fpn* of rainbow trout (GenBank: XM_021579209.2) was amplified using the cDNAs reverse-transcribed from liver RNA using the primers listed in **Table 2**. The PCR product was digested with restriction enzymes (Kpn 1and Xma 1), followed by ligation into pEGFP-N1 to construct pEGFP-Fpn. The plasmid pEGFP-Fpn was extracted from the Trans5α cells using an E.Z.N.A. Plasmid Maxi Kit (Omega). The extracted plasmid was transfected into the HEK293T cells using the TransIntro EL transfection reagents (TransGen Biotech) according to the manufacturer’s instructions. At 24 h after transfection, cycloheximide (Aladdin) was added to the HEK293T cells to give a final concentration of 75 μg/mL. After incubation for 3 h, the synthetic rainbow trout LEAPs were added to give a final concentration of 1 μM and incubated for 24 h. The changes in the position of Fpn-EGFP in the cells were investigated using a live cell station (Leica AF6000).

### Statistical analysis

The statistic *p* value was calculated using the SPSS Statistics (version 19, IBM) by one-way ANOVA with a Dunnett *post hoc* test. A *p* value < 0.05 was considered statistically significant while a *p* value < 0.01 was considered highly significant.

## Results

### Homology comparison of LEAPs

**Figure 1:**
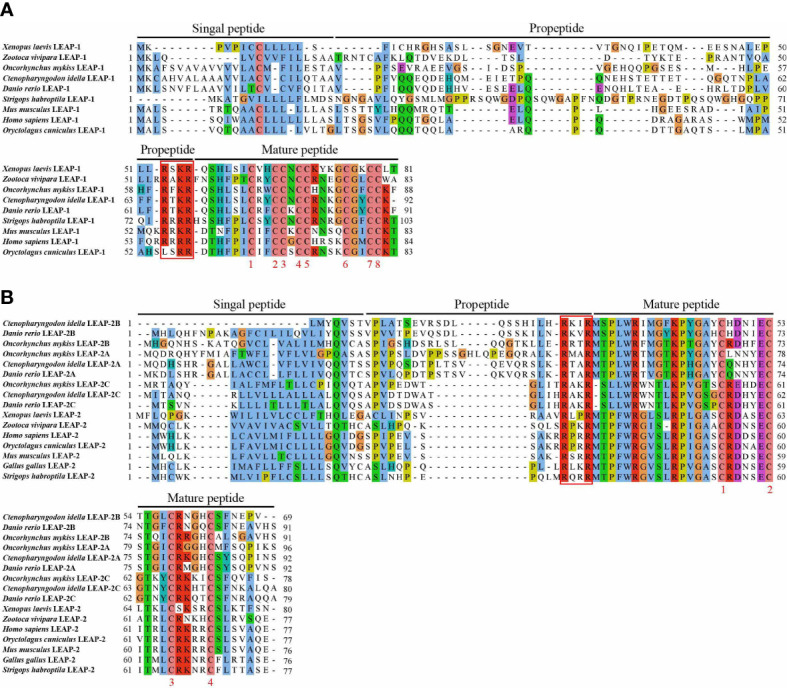
Amino acid sequence and domain organization of LEAP-1 **(A)** and LEAP-2 **(B)** from mammals, birds, amphibians, reptiles, and fish. The signal peptide, propeptide and mature peptide are denoted above the alignment. GenBank accession numbers of the selected sequences are listed in **Table 1**.

Homologous sequence alignment showed that all LEAPs consist of a signal peptide, a propeptide and a mature peptide. The sequences of the mature peptides are more conserved than those of the signal peptides and propeptides. The mature peptide of LEAP-1 and LEAP-2 possessed eight and four conserved cysteines, respectively. Interestingly, from fish to mammals, the signature of the cleavage site (RXXR) between the propeptide and the mature peptide of LEAPs are conserved in evolution **(** **Figure 1** **)**.

### Gene structure and phylogenetic analyses of LEAPs

**Figure 2:**
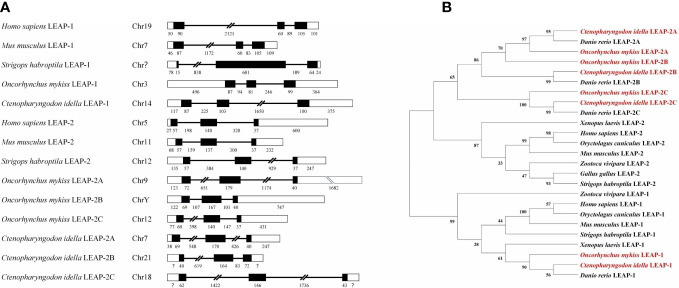
Gene structure and phylogenetic relationship of vertebrate LEAPs. **(A)** Gene structure of LEAPs from *Homo sapiens*, *Mus musculus*, *Strigops habroptila*, *Oncorhynchus mykiss*, and *Ctenopharyngodon idella*. The rectangles represent the exons, and the lines between them indicate the introns. Black and white areas indicate the coding regions and untranslated regions, respectively. The sizes of the exons and introns are shown by the numbers below. GenBank accession numbers of the selected sequences are listed in **Table 1**. The question mark means the length is unknown. **(B)** Phylogenetic relationship of LEAPs. A neighbour-joining phylogenetic tree was generated using the MEGA program with 1000 bootstrap replications. Rainbow trout and grass carp LEAPs are in red. GenBank accession numbers of the selected sequences are listed in **Table 1**.

*LEAP-1* and *LEAP-2* have the same gene structure, which is composed of 3 exons and 2 introns. The 5’ and 3’ UTR of rainbow trout and grass carp *LEAP-1* are longer than those of *LEAP-1s* from other species analyzed. The two introns of grass carp *LEAP-2C* are longer than those of *LEAP-2Cs* from other species analyzed **(** **Figure 2A** **)**.

In the phylogenetic tree constructed using the amino acid sequences of the LEAP-1 and LEAP-2 precursors **(** **Figure 2B** **)**, the LEAP-1 sequences branched apart from the LEAP-2 sequences. Teleost LEAPs were distinguished from those of other vertebrates. LEAP-2 of grass carp, rainbow trout and zebrafish contain three members, named LEAP-2A, LEAP-2B and LEAP-2C.

### Expression of *LEAPs* in rainbow trout and grass carp tissues

**Figure 3:**
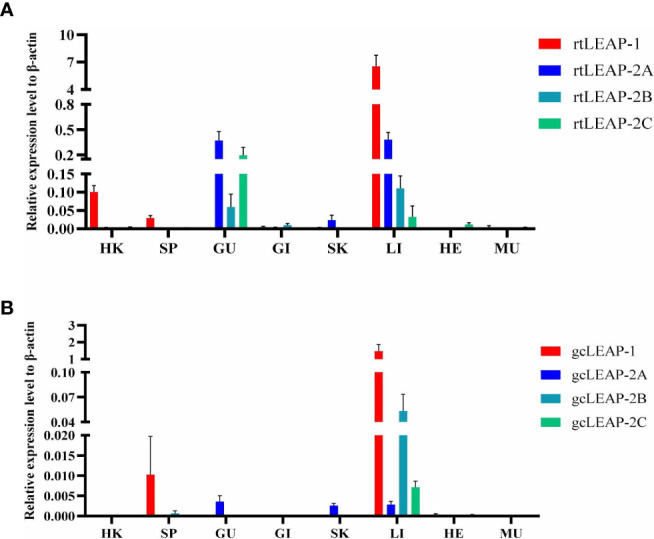
Expression patterns of rainbow trout **(A)** and grass carp **(B)** *LEAPs* in various tissues. The expression levels of *LEAPs* in lymphoid and non-lymphoid tissues were analyzed by qPCR and normalized against the expression of *β-actin* using the 2^−ΔCt^ method. Abbreviations for tissues are as follows: HK, head kidney; SP, spleen; GU, gut; GI, gill; SK, skin; LI, liver; HE, heart; MU, muscle. Data for rainbow trout represent the mean ± SEM of four individuals, and data for grass carp represent the mean ± SEM of five individuals.

The mRNA expression levels of the *LEAPs* were evaluated in several healthy rainbow trout **(** **Figure 3A** **)** and grass carp **(** **Figure 3B** **)** tissues. Rainbow trout (rt) and grass carp (gc) *LEAPs* were differentially expressed in various tissues and organs, with an overall predominance of *LEAP-1*. In general, the *LEAPs* were highly expressed in the liver and lowly expressed in the gill, heart, and muscle. *LEAP-2A* was significantly expressed in the gut and skin of rainbow trout and grass carp, and the expression of *LEAP-2C* in the gut of rainbow trout was higher than that in the liver.

### Induced expression of *LEAPs* by pathogenic bacteria

**Figure 4:**
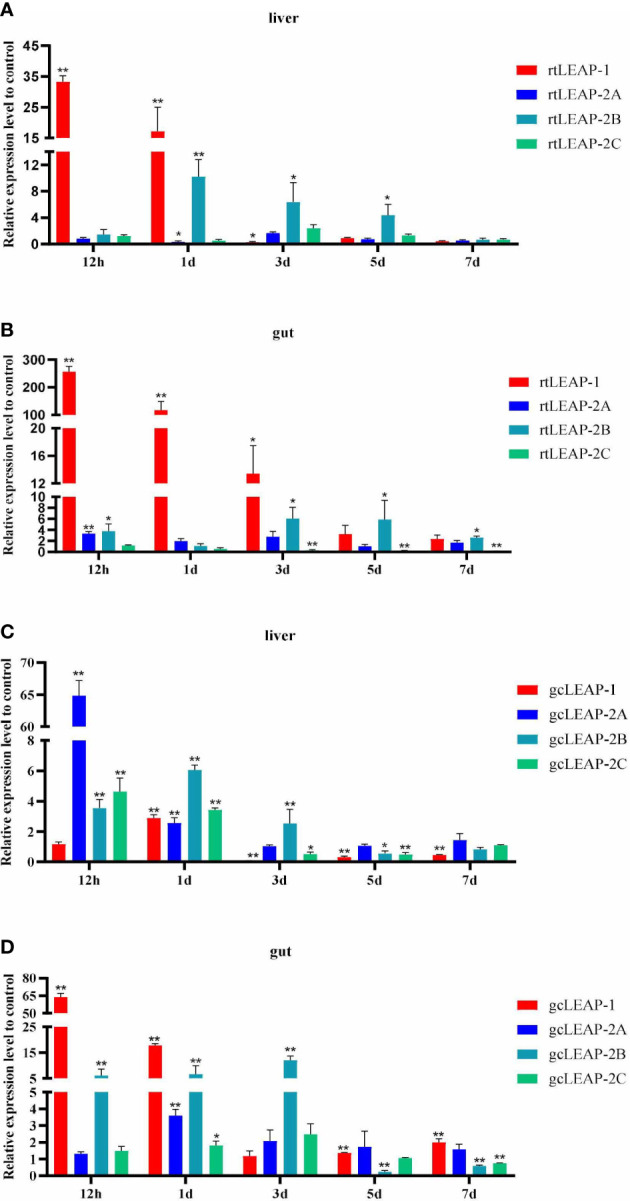
Induced expressions of *rtLEAPs* **(A, B)** and *gcLEAPs* **(C, D)** in the liver and gut under the condition of infection. Fold changes were calculated by comparing the infected group with the control group (defined as 1) using the 2^−ΔΔCt^ method. Data represent the mean ± SEM of four fish individuals. The *p* value was calculated by one-way ANOVA with a Dunnett *post hoc* test (*p < 0.05, **p < 0.01).

Expression of the total transcripts of *rtLEAP-1* and *rtLEAP-2* in the liver **(** **Figure 4A** **)** and gut (**Figure 4B**) of rainbow trout induced by *A. salmonicida* was studied. The expression level of *rtLEAP-1* increased significantly in the liver from 12 h to 1 d, and in the gut from 12 h to 3 d after infection. The expression level of *rtLEAP-2A* increased significantly in the gut at 12 h after infection. The expression level of *rtLEAP-2B* increased significantly in the liver from 1 d to 5 d, and in the gut from 3 d to 7 d after infection. However, the expression level of *rtLEAP-2C* decreased significantly in the gut from 3 d to 7 d after infection.

Expression of the total transcripts of *gcLEAP-1* and *gcLEAP-2* in the liver (**Figure 4C**) and gut (**Figure 4D**) of grass carp induced by *A. hydrophila* was studied. The expression levels of *gcLEAP-1* and *gcLEAP-2s* increased from 12 h to 3 d after infection, and then gradually returned to normal level, with *gcLEAP-2A* increased most significantly in the liver and *gcLEAP-1* increased most significantly in the gut.

### Antibacterial activities of LEAPs

**Figure 5:**
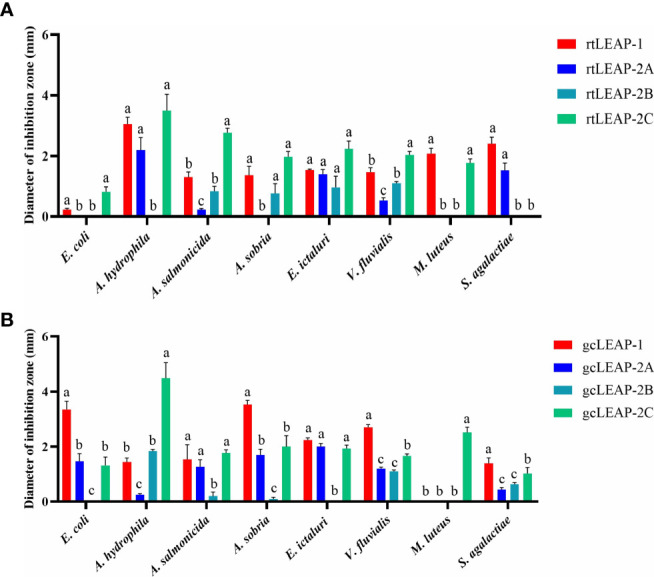
Antibacterial activities of rainbow trout **(A)** and grass carp **(B)** LEAPs against various bacteria. The underlay agarose gel containing *E. coli* ATCC25922, *A. hydrophila* XS91-4-1, *A. salmonicida* BG1, *A. sobria* CR79-1-1, *E. ictaluri* HSN-1, *V. fluvialis* WY91-24-3, *M. luteus* ATCC10240 and *S. agalactiae* ATCC13813 respectively was poured into sterile petri dishes. After the solidification, 4 mm diameter wells were punched in the agarose gel. Then 6 μL LEAP (125 μM) was added to each well, and sterile water was added as the control. Plates were incubated at the optimum bacterial growth temperature (28℃ for *A. hydrophila, A. salmonicida, A. sobria* and *E. ictaluri* or 37℃ for *E. coli, V. fluvialis, M. luteus* and *S. agalactiae*) for 3 h, and then the underlay agarose gel was covered with overlay agarose gel. The diameter of the inhibition zone around each well was measured after 24 h of incubation at the optimum bacterial growth temperature. Data represent the mean ± SEM of three independent experiments. Different letters indicate significant differences (p<0.05).

To investigate the antimicrobial properties of rainbow trout and grass carp LEAPs, we synthesized the mature peptides of rainbow trout and grass carp LEAPs and tested their antibacterial activities against a variety of bacterial strains. The results showed that all of the rainbow trout and grass carp LEAPs were antibacterial to Gram-negative (*E. coli*, *A. hydrophila*, *A. salmonicida*, *A. sobria*, *E. ictaluri*, and *V. fluvialis*) and Gram-positive bacteria (*M. luteus* and *S. agalactiae*) (**Figures 5A, B** **)**. For the same bacterium, the antibacterial activities of different LEAPs are different. Interestingly, rainbow trout and grass carp LEAP-2C showed significant inhibitory effects on *A. hydrophila*, a common and harmful pathogen in fish farming.

### Antibacterial mechanism of LEAPs

**Figure 6:**
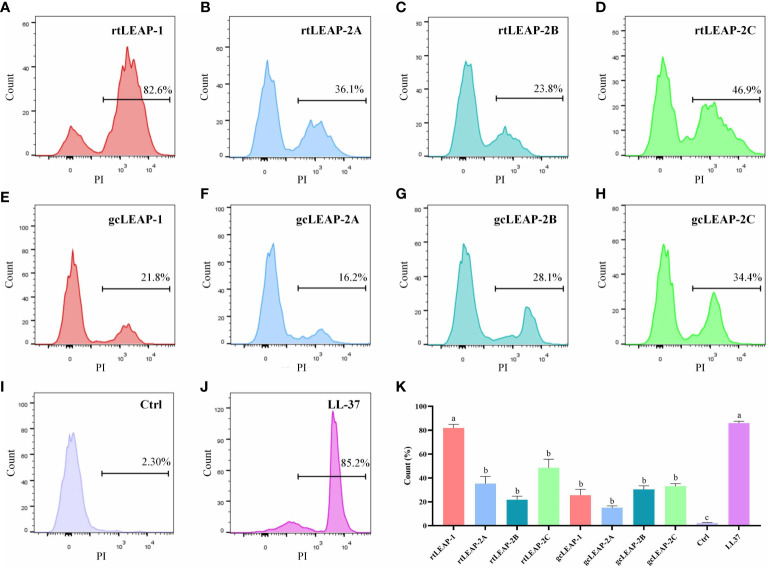
Antibacterial mechanisms of LEAPs. **(A–J)** Membrane permeability of *V. fluvialis* treated with LEAPs. The bacteria were incubated with LEAPs (8 μM) at 37°C for 1 h, and a bacterial suspension without peptide was included as a control (Ctrl). Then PI (9 μM) was added and the influx of PI was detected by flow cytometry. Data are representative results of three independent experiments. **(K)** The data in **(A–J)** were analyzed statistically. The *p* value was calculated by one-way ANOVA with a Dunnett *post hoc* test. Different letters indicate significant differences (p<0.05).

To clarify the antibacterial mechanism of LEAPs, we detected the integrity of cell membrane by bacterial membrane permeability assay. The results showed that rainbow trout and grass carp LEAPs could significantly increase the permeability of the cell membrane of *V. fluvialis* within 1 h, and rtLEAP-1 had the most significant effect, which was similar to human AMP LL-37 (**Figures 6A–K**), suggesting that LEAPs kill bacteria through membrane permeabilizing action.

### Comparison of the ability of LEAPs to internalize Fpn

**Figure 7:**
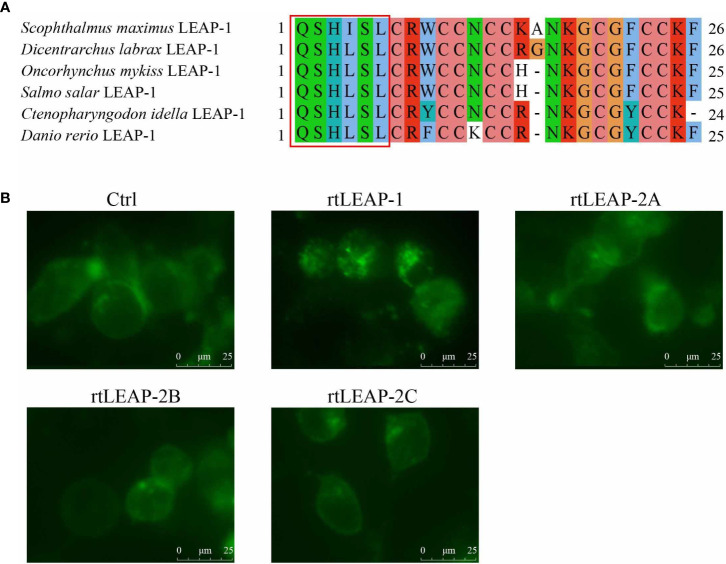
The effects of LEAPs on Fpn internalization. **(A)** Amino acid sequence alignment of LEAP-1 mature peptides in rainbow trout, grass carp, zebrafish, European sea bass, Atlantic salmon, and turbot. The Q-S/I-H-L/I-S/A-L motif at the N-terminus of the mature peptides are indicated with a red rectangle. GenBank accession numbers of the selected sequences are listed in **Table 1**. **(B)** The ability of LEAPs to internalize Fpn. HEK293T cells were seeded in 6-well plates and transfected with pEGFP-Fpn. After 24 h, cycloheximide (75 ug/mL) was added for 3 h, and then cells were incubated with rtLEAPs (1 μM) and PBS (blank control [Ctrl]) for 24 h. The images of cells were captured using a live cell station.

Through the amino acid sequence alignment of the LEAP-1 mature peptides in rainbow trout, grass carp, zebrafish, European sea bass, Atlantic salmon, and turbot, the Q-S/I-H-L/I-S/A-L motif was found in the N-terminus of the mature peptides (**Figure 7A**). To compare the ability of LEAPs to internalize Fpn, rtLEAPs were added to the cells expressing Fpn-EGFP, and the changes in the position of Fpn-EGFP in the cells were investigated using a live cell station. The results showed that only rtLEAP-1 resulted in the loss of cell surface Fpn-EGFP and the presence of intracellular Fpn-EGFP (**Figure 7B**), indicating that only rtLEAP-1 can cause the internalization of Fpn, while rtLEAP-2A, rtLEAP-2B and rtLEAP-2C cannot.

## Discussion

Just as in tetrapods, there are also LEAP-1 and LEAP-2 in fish, but the difference is that fish LEAP-1 and LEAP-2 contain multiple genes. This is most likely due to the genome duplications and positive selection in fish, which suggests that different LEAPs may perform different functions. Multiple *LEAPs* existing in fish may be contributed to the immune defense of fish, which live in a complex aquatic environment.

Through amino acid sequence alignment, it was found that the LEAP-1 sequences of rainbow trout and grass carp were similar to other species, and the identity between rainbow trout and grass carp is 87.5%. Previous studies have shown that the LEAP-2 of rainbow trout (20) and grass carp (34) contains two genes, *LEAP-2A* and *LEAP-2B*, but in the current study we found that *LEAP-2C* also exists in these two species. Phylogenetic analysis indicated that LEAP-2 has evolved separately from LEAP-1 in vertebrates, resulting in LEAP-1 having iron regulation function, while LEAP-2 doing not.

As in many fish species and other vertebrates (19, 35–37), though predominantly expressed in the liver, both *LEAP-1* and *LEAP-2* were differentially expressed in other tissues and organs of rainbow trout and grass carp. A notable phenomenon was that *LEAP-2A* was significantly expressed in the gut and skin of rainbow trout and grass carp and *LEAP-2B* and *LEAP-2C* were significantly expressed in the gut of rainbow trout. Similar results were reported in blunt snout bream, whose *LEAP-2* mRNA expression level was highest in the gut (38). Research has shown that common carp *LEAP-2A* mRNA expression level increased in the gut, gills and skin after infection (22). These suggest that LEAPs of teleost fish may play important roles in both systemic and mucosal immunity, respectively.

A significant induction of *LEAP* was observed after experimental bacterial challenge, with *LEAP-1* being the most responsive gene in the liver and gut of both rainbow trout and grass carp. In rainbow trout, *LEAP-1* expression increased significantly in the early stage of infection, and *LEAP-2B* expression increased significantly in the late stage of infection. In grass carp, the gene expression of all *LEAPs* increased after infection, and then gradually returned to normal levels. These results suggest that rtLEAPs may function for a longer period after pathogen infection to maintain robust immunity. Similar induced expression of *LEAPs* was reported in other fish species after bacterial infection. It is worth noting that the tissue expression patterns of *LEAPs* in different fish species have specificity, and different pathogens are likely to induce the expression of different LEAPs (35, 39–42). All of these studies, however, shared the same idea that multiple LEAPs in hosts perform important antimicrobial functions.

Rainbow trout and grass carp LEAPs have broad spectrum antibacterial activities against a various type of bacteria. Notably, gcLEAP-2A has antibacterial activity against several bacterial strains, including *E. coli*, which is different from the previous report that gcLEAP-2A has no antibacterial activity against *E. coli* (34). Our results suggest that the antibacterial immunity of fish was enhanced through LEAP expansion. Although LEAP-1 and LEAP-2 belong to different types, their bactericidal mechanisms are roughly the same. Rainbow trout and grass carp LEAPs kill bacteria by disrupt the cell membranes, which is similar to previous study on other species that LEAPs form multiple pairs of disulfide bonds through cysteine residues and form a stable β-sheet structure, which can destroy bacterial cell membrane, promote its permeability, and cause the leakage of cell contents and the death of bacterial cells, thus killing pathogenic bacteria (10, 43).

Previous study has shown that the expression of LEAP-1 is directly or indirectly regulated by iron storage anemia, hypoxia, inflammation, pathological conditions, cytokines, and other signals (44, 45). Under normal physiological conditions, the expression of LEAP-1 is negatively correlated with iron level (46–48). The conserved sequence Q-S/I-H-L/I-S/A-L, which is associated with iron regulation activity (49), is present at the N-terminus of the mature peptide of LEAP-1, but not LEAP-2, suggesting that LEAP-1 and LEAP-2 play different roles in iron regulation. Our results further confirm that only LEAP-1 has the ability to internalize Fpn. On the one hand, this reaction maintains the homeostasis of iron in teleost fish; on the other hand, it leads to the obstruction of iron outflow from cells and reduces the available iron content of extracellular pathogens (13, 50).

In conclusion, this study first reported the existence of the *LEAP-2C* in rainbow trout and grass carp and systematically compared the antibacterial function of LEAPs in these teleost fish, which suggested that multiple LEAPs can enhance the immunity of teleost fish through different expression patterns and different antibacterial activities to various bacteria. This enhanced our understanding of host innate immunity and provided new evidence and insights for systematically analyzing the antibacterial function of LEAPs in teleost fish.

## Data availability statement

The data presented in the study are deposited in the GenBank repository (https://www.ncbi.nlm.nih.gov/genbank/), accession numbers GQ870279.1 and OQ026323.

## Ethics statement

The animal study was reviewed and approved by the Committee on the Ethics of Animal Experiments at Huazhong Agricultural University. Written informed consent was obtained from the owners for the participation of their animals in this study.

## Author contributions

XL performed most of the experiments, analyzed most of the data, and wrote the preliminary manuscript. Y-ZH searched the *LEAP-2C* in the grass carp genome and helped with the date analysis. Y-RP helped with the gene structure analysis. JL helped with the sampling of infection experiments. Y-BJ participated in the radial-diffusion assay. Y-AZ helped with the experiment design and revised the manuscript. X-JZ designed the research, analyzed some of the data, and revised the manuscript. All authors contributed to the article and approved the submitted version.
